# State of pedestrian road safety in Uganda: a qualitative study of existing interventions

**DOI:** 10.4314/ahs.v21i3.62

**Published:** 2021-09

**Authors:** Jimmy Osuret, Stellah Namatovu, Claire Biribawa, Bonny Enock Balugaba, Esther Bayiga Zziwa, Kennedy Muni, Albert Ningwa, Frederick Oporia, Milton Mutto, Patrick Kyamanywa, David Guwatudde, Olive Kobusingye

**Affiliations:** 1 Department of Disease Control and Environmental Health, School of Public Health, College of Health Sciences, Makerere University, Kampala, Uganda; 2 Department of Epidemiology and Biostatistics, School of Public Health, College of Health Sciences, Makerere University, Kampala, Uganda; 3 School of Health Sciences, Kampala International University, Bushenyi, Uganda; 4 Department of Epidemiology, University of Washington, Seattle, WA, USA

**Keywords:** Pedestrian, safety intervention, qualitative, Uganda

## Abstract

**Background:**

Pedestrians in Uganda account for 40% of road traffic fatalities and 25% of serious injuries annually. We explored the current pedestrian road traffic injury interventions in Uganda to understand why pedestrian injuries and deaths continue despite the presence of interventions.

**Methods:**

We conducted a qualitative study that involved a desk review of road safety policy, regulatory documents, and reports. We supplemented the document review with 14 key informant interviews and 4 focus group discussions with participants involved in road safety. Qualitative thematic content analysis was done using ATLAS. ti 7 software.

**Results:**

Five thematic topics emerged. Specifically, Uganda had a Non-Motorized Transport Policy whose implementation revealed several gaps. The needs of pedestrians and contextual evidence were ignored in road systems. The key programmatic challenges in pedestrian road safety management included inadequate funding, lack of political support, and lack of stakeholder collaboration. There was no evidence of plans for monitoring and evaluation of the various pedestrian road safety interventions.

**Conclusion:**

The research revealed low prioritization of pedestrian needs in the design, implementation, and evaluation of pedestrian road safety interventions. Addressing Uganda's pedestrian needs requires concerted efforts to coordinate all road safety activities, political commitment, and budgetary support at all levels.

## Background

More than 1.35 million deaths and up to 50 million injuries occur on the world's roads annually^1^. The burden of road traffic deaths and injuries is more pronounced among vulnerable road users - pedestrians, cyclists, and motorcyclists especially those living in low- and middle-income countries (LMICs)^1^–^3^. Vulnerable road users are neglected in road safety management programs in many countries, and yet they account for more than half of the global road traffic deaths^1^. Moreover, between 2013 and 2016, no reductions in road traffic deaths were observed in low-income countries (LICs)^1^. The road traffic death rate in Uganda is 29 deaths per 100,000 population, which is higher than the global estimate of 18 deaths per 100,000^1^. Pedestrians comprise the largest group of road users killed in Uganda, accounting for 40% of fatalities and 25% of serious injuries^4^.

Many LMICs are lagging behind in road safety and there is pressure to address the problem of road traffic crashes among vulnerable road user^1^. This is due to the limited action taken on pedestrian road safety leaving an implementation gap^5^. The biggest challenge to implementation is limited budget support that does not allow for road safety in many LMICs^5^. Strategies to reduce pedestrian road traffic crashes that have been used globally include speed management, enforcement, pedestrian infrastructure treatment, visibility programs and awareness campaigns^6^. However, the most successful strategies are those implemented holistically and embrace the interaction of the vehicle, roads and road user as a system^7^.

Uganda has a legal framework that underpins pedestrian road safety management under the Non-Motorized Transport Policy, National Road Safety Policy, and the Traffic and Road Safety Act 1998. Interventions to reduce pedestrian road traffic injuries (RTIs) in Uganda include pedestrian sidewalks, overpasses, traffic calming mechanisms, road safety campaigns, police enforcement, and road safety educational programs^4^. There is growing recognition of the road traffic injury burden in Uganda^8^–^11^. As a result, substantial research has been generated on RTI determinants^12^–^14^, estimation of pedestrian injury burden^15^–^17^, the road traffic injury distribution^18^ and road safety measures^19^–^21^. However, there is a dearth of research on understanding why pedestrian injuries and death continue. Addressing Uganda's pedestrian road safety needs requires an understanding of contextual factors related to the policy environment, intervention implementation challenges and opportunities. Considering this, the study explored the current pedestrian RTI prevention interventions in Uganda to understand the design, implementation and evaluation aspects, and why the burden persists despite the interventions.

## Methods

### Study design

We conducted a qualitative study in 2018 that was based on the constructivism paradigm which recognizes the subjective creation of meaning^22^. A qualitative study design offered an opportunity to triangulate information and gather a deep understanding of challenges and opportunities during the design and implementation of interventions to reduce pedestrian RTIs. The study comprised three primary methods; document review, key informant interviews (KIIs) and focus group discussions (FGDs).

### Study setting

Participants were selected from the Kampala Metropolitan area which contributes nearly half (49%) of the road traffic crashes in Uganda^4^. It covers all of Kampala city, and parts of surrounding Wakiso and Mukono districts with populations that range from 862,701 to 2,548,000^23^. Uganda'spopulation was 39 million as of the 2018 National population census estimates^24^. Walking is a dominant mode of transport^25^. Other common modes of transportation include use of private cars, commercial motorcycles (“boda bodas”), commuter mini buses, and cycling^25^. Kampala Metropolitan area has paved and unpaved road infrastructure^9^. The city roads are under the management of Kampala Capital City Authority (KCCA) and national roads under the Uganda National Roads Authoity (UNRA)^9^. The Uganda Police is responsible for enforcement of traffic laws and regulations^4^.

### Sampling and data collection

**Figure 1:**
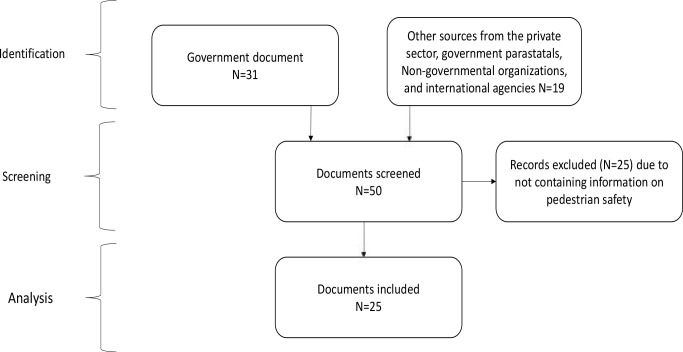
Flow diagram illustrating the document selection process

The document review included road safety policy documents and research activity reports from the government, the private sector, government parastatals, non-governmental organizations (NGOs), and international agencies working on road safety. Documents for review were mainly provided by key informants in hardcopy. Softcopies documents were downloaded from websites of relevant institutions involved in road construction, road safety policy formulation, enforcement and advocacy in Uganda. Only documents with sections relevant to pedestrian road safety were included in the study (Figure 1). Source documents selected were obtained from Insurance Regulatory Authority^26^; Kampala Capital City Authority (KCCA)^27^; Ministry of Lands, Housing and Urban Development (MoLHUD)^25^; The Parliament of Uganda^28^; Uganda Police Force (UPF)^29^, ^30^; Ministry of Works and Transport (MoWT)^31^–^40^; Uganda National Roads Authority (UNRA)^41^, ^42^; Safe Way Right Way^43^–^48^; United Nations^9^. An inventory of all included documents was created for tracking purposes. Two reviewers then independently extracted pedestrian safety data on existing plans, policies, interventions and programs from all included documents using structured data extraction forms. Additionally, we extracted data on aspects of leadership, stakeholder engagement, implementation processes, monitoring and evaluation indicators.

Interviews were conducted with 14 purposively selected key informants using an interview guide. The key informants were drawn from stakeholders involved in pedestrian safety of whom 2 were female and 12 were male. These included representatives from the ministries of Works and Transport; Lands, Housing, and Urban Development; Health; the Uganda police directorate of traffic and road Safety; the National Road Safety Council; KCCA; Parliament; and road safety NGOs. We conducted 4 homogeneous focus group discussions with one group each for pedestrians, commuter taxi drivers, boda-boda drivers, and private car drivers. A total of 32 participants 18 years and above were interviewed in the FGDs of whom 25% were female and 75% male. Focus group participants were purposively selected from areas of heavy traffic and pedestrian concentrations (e.g. around markets) to represent the key road users. The guides for the KIIs and FGDs were pretested and adapted based on feedback before data collection. Data were collected by the investigators and trained research assistants. The interview and focus group guides covered aspects of pedestrian safety interventions, the impact of interventions, stakeholders involved in pedestrian safety, factors associated with pedestrian injuries and deaths, and challenges impeding implementation. The interviews and discussions were audio-recorded after seeking permission from the participants, and field notes taken. Probes were applied based on the responses of the participants. We conducted key informant interviews and focus group discussions until no new information was attained.

### Data management and analysis

#### a Document review

For the document review, a harmonized summary was created through consensus between the two reviewers, and where there were still areas of disagreement, a third reviewer was consulted. The data from the document analysis supplemented the findings from the interviews.

#### b Interviews

The KIIs and FGDs were transcribed verbatim and cleaned. Where discussions were held in Luganda (local language in the study area), the transcripts were translated into English by a language specialist in preparation for analysis. The transcripts were exported to ATLAS.ti Version 7 software tool for coding and analysis. Rigour was enhanced through triangulation where two groups independently coded, analysed and then compared the findings. We used a qualitative thematic content analysis approach^49^,^50^ where categories and themes inductively arose from the data.

### Ethics considerations

Ethical clearance was obtained from Makerere University School of Public Health Research and Ethics Committee (approval #469) and the Uganda National Council for Science and Technology (registration #SS 4319). We obtained informed and written voluntary consent from all participants. All data obtained during the study were treated with confidentiality by removing any personal data that would trace back to the study participant.

## Results

We explored the current pedestrian road traffic injury interventions in Uganda to understand aspects of the design, implementation, programmatic challenges and monitoring and evaluation. The results are presented in five thematic topics from the data analysis (table 1)

**Table 1:** Emerging themes from the Desk review, key informant interviews, and focus group discussions

CODES	THEMES
Pedestrian safety guidelines, rules and regulations	Design of pedestrian RTI interventions
The frequent occurrence of pedestrian road traffic crashes prompted the establishment of pedestrian safety measures Heavy pedestrian traffic areas like markets and schools Initiatives by NGOs and external funding to establish pedestrian safety measures	Implementation of pedestrian RTIs interventions
Gaps in implementation of pedestrian road safety policies inadequate pedestrian facilities encroachment of pedestrian facilities by motorists	Implementation of road safety policies
Inadequate funding Low priority for pedestrian safety Inadequate political support Lack of collaboration among stakeholders Limited community engagement in pedestrian safety Weak institutional capacity	Programmatic challenges of pedestrian RTIs interventions
No evidence of plans for monitoring and evaluation for various pedestrian RTI interventions Poor crash data systems to monitor and evaluate pedestrian crashes	Monitoring and Evaluation of pedestrian RTI interventions

### Design of pedestrian RTI interventions

Guidelines such as the National Physical planning and General specifications of roads^25^, ^42^ were sometimes used in the design of pedestrian RTI interventions. These guidelines have specifications for road user behaviour e.g. following all traffic rules^30^. Some of the key informants affirmed to the fact that they use the existing guidelines in designing interventions.

...*usually when we are considering the planning we use what we call the physical planning standards. With these standards we consider the size of the road; that the road should be of this size. And within the sizes of those roads we know that this road is in position to cater for a carriageway, to cater for services and infrastructure, and even to cater for the pedestrians' walkways and so on, depending on the available planning*. Key informant

### Implementation of pedestrian RTI interventions

There was a consensus from the interviews that the main driving force for the implementation of pedestrian safety measures was pressure following the frequent occurrence of pedestrian road traffic crashes and fatalities at particular spots.

....*pedestrian road safety measures were put after primary school children were knocked. Three children were knocked and they died on spot and that is when a decision was made to put the measures. Right now there is a crossing guard to help them (children) cross which was not the case before*. FGD participant

...*you are aware that last year alone (2017) 40% of the people that died were pedestrians. Sosic as implementers we have to think about how tosic reduce this burden*. Key informant

Another factor that influenced the implementation of interventions to reduce pedestrian RTIs was the presence of heavy pedestrian traffic and volumes in areas like schools, markets, or hospitals. The presence of these institutions or business areas was a driving force for implementing road safety measures e.g. traffic calming to protect pedestrians.

*These (speed humps) at schools were put there to help the children who cross while going and coming back from school*. FGD participant

Efforts by NGOs (e.g. Uganda Red Cross Society) whose mandate include road safety, and the availability of external funding from international agencies or oil companies influenced the implementation of pedestrian safety measures to reduce RTIs

*There are several other NGOs, other private sector agencies e.g. Safe Way Right Way, VIVO energy, Total, Tullow Oil, and so many others. So the private sector is involved and does a lot of work as well in road safety. The United Nations Economic Commission for Africa recently funded the performance review for Uganda but through the Ministry of Works and Transport*. Key informant

### Implementation of road safety policies

The two policies analysed included the non-motorized transport policy (NMTP) and the National Road Safety policy. These policies provided for the incorporation of Non-Motorized Transport infrastructure for pedestrians^37^. Walking was to be recognized in transport planning, design and infrastructure provision^37^. These policies further promoted pedestrian safety and called for the development of suitable medium-distance pedestrian and bicycle routes with appropriate infrastructure^37^. However, analysis of these policies revealed several gaps, evidenced by the inadequate safe walking and crossing facilities^9^. Most roads were designed and constructed without considering the needs of pedestrians and other non-motorized modes of transport according to key informants. The existing pedestrian facilities were encroached on by motorists and vendors according to FGD participants.

*Pedestrian facilities are occupied by either street vendors or by parked vehicles and therefore pedestrians end up walking in the middle of the road*. FGD participant

### Programmatic challenges of pedestrian RTIs interventions

We identified policy challenges as well as gaps in the process of designing and implementing interventions to reduce pedestrian RTIs in Uganda. Uganda had a road fund according to the document review^9^, but there were no clear budget lines to support the implementation of pedestrian road safety measures by line ministries and road agencies. Inadequate funds and road maintenance backlogs limited the capacity of road safety institutions to effectively carry out pedestrian safety activities^9^. Uganda traffic police had inadequate finances and human resources to effectively enforce road safety measures, according to key informants.

...*funding is the challenge because if for example, you are building a road with a raised walkwayhmmm it would be more expensive than a road with an open way ......, because for a raised walkway you have to buy these pipe culverts burry them in the ground, construct the curbs, raise the walkway, so it's much more expensive*. Key informant

Road safety was a low priority in road designs countrywide due to the inadequate political and technical support in lobbying for road safety financing. We found several road safety guidelines in place, but vulnerable road users received inadequate consideration during planning and resource allocation for road safety interventions^9^. Roads were designed and constructed without considering the needs of pedestrians and other non-motorized modes of transport. Some of the focus group participants mentioned that the roads were poorly maintained, lacked pedestrian crossings and markings, and delays were reported in carrying out periodic maintenance works. In some areas, roads were reported to be narrow with inadequate safe walking facilities.

*Government priority for road safety is still low. Let me tell you, about 30 or more people died last week in crashes. If these were from nodding disease, Parliament would be up in arms for money for nodding disease*. Key informant

*There are planners who think that roads are for vehicles and there are some designers who design with the thinking that roads are for vehicles only*. Key informant.

We found several stakeholders including the Kampala Capital City Authority, Ministry of Health, Uganda National Roads Authority, the private sector, NGOs, international organizations that were directly or indirectly involved in pedestrian safety. However, there was lack of coordination and collaboration among these stakeholders because of the lack of a concrete multi-sectoral action plan to coordinate all road safety activities. In some instances, there was duplication of interventions among various road safety stakeholders.

*The challenge we get is that some of the interventions are not coordinated (hmmm) so you have this onestakeholder doing something similar to another, so the programs are not coordinated. They all compete for visibility*. Key informant

There was insufficient community involvement in planning and implementing pedestrian road safety interventions as reported from the focus group discussion. Some interventions were implemented without community participation and consultation and this negatively affected their adoption.

*There is a flyover which was put at Nakawa for pedestrians to use but since they were not sensitized about its importance, they don't use it; they all use the road. The same applies to the Kalerwe roundabout, the pedestrians use the road yet a flyover is there, but generally, it was not well positioned, it would have been (better) near the market. Commuter taxi driver*. FGD participant.

The pedestrian facilities were encroached on by other activities like street vending, parking and motorists who drive on the few available pedestrian walkways^9^. Competition for the limited space continued to put pedestrians at risk.

*Our roads are narrow and congested. For instance, there is a mixing of hawkers, boda-boda riders, someone is crossing and as you try to avoid a pothole, and you knock pedestrians. Commuter taxi driver*. FGD participant.

The document review revealed a weak institutional framework and low capacity at almost every level, and this hindered implementation of many policies and regulations^9^. Besides financial limitations, insufficient equipment and personnel hindered the implementation and enforcement of pedestrian safety interventions.

### Monitoring and Evaluation of pedestrian RTI interventions

There was no evidence of plans to measure the effectiveness of pedestrian road safety interventions according to key informants. There were no clear goals, indicators, or targets to measure the process and impact of pedestrian interventions implemented. There were no national targets for the reduction of pedestrian road traffic deaths.

...*there is quite some work to do in that area, we don't have very robust monitoring and evaluation. All we know is that when we carry out an intervention we get some feedback from the public that now the danger has been averted*. Key informant

Police and health sector data were the main sources used to estimate the road traffic burden in Uganda. However, data systems did not provide useful information to monitor and evaluate pedestrian RTI interventions. This is because the existing data is limited in the information captured and do not provide a true estimate of the burden of road traffic crashes, injuries, deaths, and their economic impact^16^.

*We don't have quantitative data to estimate the actual number of crashes every year. That evidence is not there.* Key informant

## Discussion

In this qualitative study, we explored the current pedestrian road traffic injury interventions in Uganda to understand the design, implementation and evaluation aspects. The desk review revealed the presence of guidelines, rules and regulations that had useful information for pedestrian road safety interventions. However, we found low prioritization of pedestrian needs in road system planning and designing, and this is consistent with findings from other LICs^51^, ^52^. Integrating road safety and urban mobility strategies in the planning and design of the road systems in Bogota Colombia reduced pedestrian traffic death by half^1^. Research suggests that design standards that meet the needs of vulnerable road users reduce pedestrian injuries and death^51^. This has implications for road safety planning in Uganda to take into account pedestrian needs.

The main driving factors for the implementation of pedestrian RTI interventions in Uganda were the occurrence of pedestrian road traffic fatalities at a particular spot; and the presence of heavy pedestrian volumes. This is because of the low uptake of road safety research evidence in Uganda and many LICs^52^. This explains the limited success in the reduction of pedestrian RTIs and death in Uganda. On the contrary, pedestrian RTIs in high-income countries (HICs) are addressed through implementation of evidence-based interventions^53^. Uganda could benefit if she implements the most appropriate measures to reduce pedestrian RTIs and death.

The key programmatic challenges in the implementation of policies and the pedestrian RTI interventions included inadequate funding, lack of political support, and lack of stakeholder collaboration. The major barrier to the implementation of pedestrian safety interventions and policies in Uganda and LICs is the lack of clear dedicated funds^5^, ^54^. HICs have funds set aside for pedestrian road safety projects^1^. Road safety in LICs competes with other priorities such as tuberculosis, malaria, and HIV^5^. Furthermore, the return on pedestrian road safety infrastructure investment is low and takes much longer^1^. The limited funding to road safety makes it challenging to address the institutional capacity in terms of human resources^5^. Therefore road safety agencies in LICs look to external funding sources^52^ that tend to prioritize road systems for vehicles and so pedestrians become exposed to dangerous roads.

Most roads in Uganda were constructed without considering the needs of pedestrians and other non-motorized modes of transport. The lack of political will was a hindrance to the implementation of measures to address pedestrian RTIs and this has been observed in other LMICs^55^. Prioritising road safety in the political agenda of Spain reduced the number of RTIs^56^. Political buy-in ensures prioritization of pedestrian road safety at national level planning and budgetary allocation^5^. This is because governments are the key drivers in response to RTIs^5^. Achieving sustainable pedestrian road safety requires a political commitment that is demonstrated through effective government leadership and ownership^5^, ^7^. Therefore there should be efforts by road safety advocates for the integration of pedestrian road safety into the Uganda national plans.

Lack of stakeholder collaboration and community participation emerged as a gap in the implementation of pedestrian RTI interventions. This is because road safety is the sole responsibility of a single sector and this affects the implementation and utility of pedestrian road safety measures^55^. A case study of a successful collaboration in Malaysia was used to engage policy makers and stakeholders in road safety research^57^. Countries that have addressed pedestrian RTI involve many sectors (e.g. government, civil society, non-governmental organizations, transport, police, health, community, and communication) that have critical actions to perform^7^. Therefore, the lead road safety government institution in Uganda should develop a multi-sectoral road safety strategy to coordinate all road safety stakeholders and should have clear targets and funding^7^.

Monitoring and evaluation are critical to determine the effectiveness of pedestrian RTI interventions and also for evidence-informed decisions for policy, practice and political buy-in^5^. European countries use road safety decision support systems to support evidence-based policy making for road safety^6^. However, no attention was paid to the monitoring and evaluation of pedestrian RTI interventions in Uganda. LICs often report on the interventions implemented, but not on the progress and impact of pedestrian RTI interventions. The existing data on pedestrian crashes do not provide a true estimate of the burden to enable monitoring and evaluation^16^, ^54^.

One of the limitations of the study is that we did not use a comprehensive search strategy to identify the documents included in the review and might have missed out some literature on pedestrian safety in Uganda. However, we attempted to address this by supplementing the desk review with a qualitative component to obtain thick descriptions, utilising key informant interviews and focus groups with people knowledgeable and involved in pedestrian safety.

## Conclusion

The research revealed very low prioritization of pedestrian needs in the transport system. This had translated into gross neglect in the design, implementation, and evaluation of interventions to reduce pedestrian RTIs in Uganda. The key programmatic challenges in the implementation of policies and the pedestrian RTI interventions included inadequate funding, lack of political support, and lack of stakeholder collaboration. Interventions were not evidence-based and there was no evidence of plans for monitoring and evaluation of pedestrian RTI interventions. Addressing Uganda's current pedestrian needs requires concerted efforts to coordinate all road safety activities, political commitment, and budgetary support.

## Data Availability

All data generated or analysed during this study are included in this published articleand its supplementary information files. We removed identifiers from the transcripts for confidentiality.
